# Non-parametric estimation of survival in age-dependent genetic disease and application to the transthyretin-related hereditary amyloidosis

**DOI:** 10.1371/journal.pone.0203860

**Published:** 2018-09-25

**Authors:** Flora Alarcon, Violaine Planté-Bordeneuve, Malin Olsson, Grégory Nuel

**Affiliations:** 1 Mathématiques appliquées Paris 5 (MAP5) CNRS: UMR8145 – Université Paris Descartes – Sorbonne Paris Cité, Paris, France; 2 Hôpital Universitaire Henri Mondor, Département de Neurologie Créteil, France; 3 Inserm, U955-E10, Créteil, France; 4 Umea university, Norrlands university hospital, NUS M31, Umea, Sweden; 5 Institute of Mathematics (INSMI), National Center for French Research (CNRS), Paris, France; 6 Laboratory of Probability (LPMA), Université Pierre et Marie Curie, Sorbonne Université, Paris, France; Edith Cowan University, AUSTRALIA

## Abstract

In genetic diseases with variable age of onset, survival function estimation for the mutation carriers as well as estimation of the modifying factors effects are essential to provide individual risk assessment, both for mutation carriers management and prevention strategies. In practice, this survival function is classically estimated from pedigrees data where most genotypes are unobserved. In this article, we present a unifying Expectation-Maximization (EM) framework combining probabilistic computations in Bayesian networks with standard statistical survival procedures in order to provide mutation carrier survival estimates. The proposed approach allows to obtain previously published parametric estimates (*e.g.* Weibull survival) as particular cases as well as more general Kaplan-Meier non-parametric estimates, which is the main contribution. Note that covariates can also be taken into account using a proportional hazard model. The whole methodology is both validated on simulated data and applied to family samples with transthyretin-related hereditary amyloidosis (a rare autosomal dominant disease with highly variable age of onset), showing very promising results.

## Introduction

In monogenic diseases with variable age of onset, an accurate estimation of the survival function for the mutation carriers is essential. Since potential factors (*e.g.* genetic or environmental factors) can modify this age of onset, it is important to identify these factors and estimate their effects. These estimations are then usually combined into a proportional hazard model that is typically used to provide individual risk assessment as well as to establish prevention strategies.

In the context of genetic diseases with variable age of onset, geneticists usually focus on the *penetrance* function, that is the cumulative risk of being affected by a given age for mutation carriers defined by

$$\begin{array}{c} F(t)=P(\text{the}\;\text{disease}\;\text{is}\;\text{diagnosed}\;\text{before}\;\text{age}\;t) \end{array}$$

as the age-specific cumulative distribution function of the waiting time to disease diagnosis [1–3]. Since in this paper one aims at exploiting standard statistical survival analysis, we will rather consider the *survival* function defined by:

$$\begin{array}{c} S(t)=P(\text{the}\;\text{disease}\;\text{is}\;\text{not}\;\text{diagnosed}\;\text{before}\;\text{age}\;t) \end{array}$$

However it is straigthforward to obtain the penetrance function from the survival one (and conversely) since *F*(*t*) = 1 − *S*(*t*). In order to avoid any confusion, please note that the survival function considered here corresponds to the *cause-specific* survival (disease diagnosis) and not to the *overall* survival. We do not consider any competing risk in the present work, and censoring (*e.g.* death) is always assumed to be independent from the waiting time of interest. Note that for severe disease (*e.g.* cancer), death is often affected by the disease status, but since this event usually occurs after diagnosis, which does not affect our model.

When estimating mutation carrier survival, the main challenge comes from the fact that most genotypes are not observed. Taking into account this uncertainty is then slightly different depending on whether the disease has sporadic cases or not. In complex diseases with monogenic sub-entities, in which only a minority of cases is due to rare mutations (*e.g.* breast cancer with BRCA mutations [4–6]) both non-carriers and mutation carriers might be affected. It is therefore necessary to provide a survival function for non-carrier which is typically obtained from the general population. In monogenic diseases such as the hereditary Tranthyretin Amyloidosis (hATTR) [2], all affected individuals are necessary carriers and thus, the disease incidence among non-carrier is equal to zero. Nevertheless the problem remains challenging since a non-affected individual at age *t* might either be a non-carrier or a carrier who “survived” until age *t*. For the sake of simplicity, we only consider in this article the monogenic diseases case; however the suggested method is straightforward to extend to complex diseases with monogenic sub-entities as long as the incidence or survival among non-carriers is available.

In the last decades, several methods have been proposed for estimating the penetrance or survival functions from pedigrees (see *e.g.*, [1–3, 6]). All these methods rely on a parametric model, namely the Weibull function, to describe the penetrance function. In these papers, unknown genotypes are handled through the Elston-Stewart algorithm [7] and likelihood function is maximized with *ad hoc* implementations [8]. Probably due to their complexity, the resulting methods were never made publicly available and were therefore scarcely used. The main objective of this paper is to provide a unified and flexible publicly available methodology that can both provide a stable implementation of the previously published parametric estimators and more general non-parametric estimates. Such estimates were previously considered in [9] but only in the non-realistic case where all genotypes were observed.

In order to achieve this objective, we reformulate the problem in the Expectation-Maximization (EM) framework [10] which provides a general iterative algorithm for optimizing the likelihood of any statistical model with partially missing data (here the unobserved genotypes). In the EM algorithm we alternate two main steps: the E-step where we compute individual weights as posterior mutation carrier distributions using the current estimates; and the M-step where we update the estimates using the observations and the weights computed at the E-step. Unlike previous works [1–3, 6] we do not want to provide an *ad hoc* implementation of these two steps but rather taking advantage of well established and robust procedures. We use probabilistic computations in Bayesian networks for the E-step [11], and classical survival analysis methods for the M-step [12].

Our method can be either used with parametric estimation like previously done in the literature (*e.g.* Weibull or exponential waiting time distribution, etc.) or with non-parametric approaches (*e.g.* Kaplan-Meier or Nelson-Aalen). To the best of our knowledge, this is the first time that a non-parametric method estimate penetrance function with unknown genotype is proposed.

The paper is organized as follows: Section “Methods” contains the main contribution of this paper which includes the model formulation, the EM-framework and the detailed E- and M-steps. Then, Section “Validation on Simulated Datasets” presents several simulation analyses that validate the method while Section “Application to the hATTR” applies the proposed method to hATTR families from different origins (French, Portuguese, and Swedish). Finally, some conclusions are drawn in Section “Discussion”. A minimal R [13] source code demo is provided as supplementary material.

## Methods

This section is devoted to the description of the proposed methodology. The objective is to estimate the cause-specific survival function for individuals carrying the disease mutation. We first introduce the model decomposed into a genetic-specific part and a survival-specific part. Then we present the EM framework and detail both the E-step using belief propagation in Bayesian networks and the M-step using existing tools from the survival analysis community.

### The Model

Let us consider *n* individuals in set I={1,…,n}. We denote by F⊂I the subset of founders (i.e. individuals without ancestors in the pedigree) and we denote by I\F the set of non-founders (i.e. individuals with ancestors in the pedigree). Let us denote by ***X*** = (*X*_1_, …, *X*_*n*_) ∈ {00, 01, 10, 11}^*n*^ the genotypic random vector defined such as *X*_*i*_ is the genotype of the individual *i*. The first entry (respectively the second entry) represents the number of paternal (resp. maternal) disease alleles. For instance *X*_*i*_ = 01 means that the individual *i* carries the mutation, is heterozygous and that his mutation has been transmitted by his mother. Also, we denote by $X_{{\text{pat}}_{i}}$ (resp. $X_{{\text{mat}}_{i}}$) the paternal (resp. maternal) genotype of any non-founder individual i∈I\F. Let us remind that the vector ***X*** is partially observed; first because individuals are rarely genotyped, secondly because the parental transmission pattern is only indirectly observed through the family relationship. Therefore, unobserved genotypes will be estimated according to genotypic information on the whole pedigree (see Section “E-step”). We denote by $T=\left(T_{1},\ldots ,T_{n}\right)\in R^{n}$ the random vector defined such as *T*_*i*_ is the time at diagnosis if the individual *i* is affected by the disease (i.e. *δ*_*i*_ = 1) while *T*_*i*_ is the time at last follow-up (censoring) if the individual *i* is not affected (i.e. *δ*_*i*_ = 0); where ***δ*** ∈ {0, 1}^*n*^ is the censoring indicator. Finally, the model can be written as follows:

$$\begin{array}{c} P\left(X,T\right)=\underset{\text{genetic}\;\text{part}}{\underset{︸}{P(X)}}\times \underset{\text{survival}\;\text{part}}{\underset{︸}{P(T|X)}} \end{array}$$

where P(X,T) denotes the joint probability distribution of ***T*** and ***X*** and P(T|X) denotes the conditional distribution of ***T*** given ***X***.

As an example, let us consider a simple nuclear family defined by two ancestors and three children. In Table 1, the first column corresponds to the index *i* of the individual, the second one to the paternal index (with the convention that we use 0 for founders), the third one to the maternal index (0 for founders), the fourth one to the censoring indicator (*δ*_*i*_ = 1 if the individual *i* is affected and *δ*_*i*_ = 0 if not), the fifth one to the time *T*_*i*_ and the last one to the genotype *X*_*i*_.

**Table 1:** Example: A simple nuclear family.

*i*	pat_*i*_	mat_*i*_	*δ*_*i*_	*T*_*i*_	*X*_*i*_
1	0	0	1	45	01
2	0	0	0	64	00
3	1	2	0	25	00
4	1	2	0	31	10
5	1	2	0	36	00

*i is the individual index, pat*_*i*_ *the paternal index (*0 *for a founder), mat*_*i*_ *the maternal index (*0 *for a founder*), *δ*_*i*_ *the event indicator (*0 *if unaffected at age T*_*i*_, 1 *if affected at age T*_*i*_), *T*_*i*_ *is the observed age either at last follow-up* (*δ*_*i*_ = 0) *or at disease diagnosis* (*δ*_*i*_ = 1), *X*_*i*_ ∈ {00, 01, 10, 11} *is the genotype*.

#### Genetic part

We assume the Mendelian transmission of the alleles and the Hardy-Weinberg distribution of the founder’s alleles with allele frequency *f*. This means that for any founder i∈F we have $P\left(X_{i}=00\right)={\left(1-f\right)}^{2}$, $P\left(X_{i}=01\right)=P\left(X_{i}=10\right)=f\left(1-f\right)$, and $P\left(X_{i}=11\right)=f^{2}$. In the case where the survival function for carriers is fully non-parametric (see Section “Survival Part”), the frequency *f* is non identifiable since the survival for carrier can easily account for any arbritrary mixture of carrier and non-carriers. This is a classical problem arising with mixture with non parametric components. A classical solution to this problem is to consider instead parametric components whose more constrained nature prevent identification issues (see [14] in the FDR context, and [15] in the survival context with cure models).

We will hence either assume that *f* is known (which is quite such genetic disease—*e.g.* BRCA mutations in breast cancer), or, in the extreme situation where this information is unknown, we will use a parametric model (*e.g.* Weibull, Gaussian, logistic, etc. [16]) to fit this parameter as a prior step before refining survival estimates using our non-parametric approach. Thus, the genetic part can be written as follows:

$$\begin{array}{c} P(X)=\prod_{i\in F}P\left(X_{i}\right)\prod_{i\in I\backslash F}P(X_{i}|X_{{\text{pat}}_{i}},X_{{\text{mat}}_{i}}) \end{array}$$

Since the *n* individuals might belong to completely independent families, it is clear that the genetic likelihood function can be computed separately on these independent families. However, the notations are still valid but simpler by combining all families into a single pedigree file.

As an example, let us compute this probability for the family of Table 1 where the observed genotypic vector is ***x*** = (01, 00, 00, 10, 00):

$$\begin{array}{ccc} P(X=x) & = & P(X_{1}=01,X_{2}=00,X_{3}=00,X_{4}=10,X_{5}=00)\\ & = & P\left(X_{1}=01\right)\times P\left(X_{2}=00\right)\times P\left(X_{3}=00|X_{1}=01,X_{2}=00\right)\\ & & \times P\left(X_{4}=10|X_{1}=01,X_{2}=00\right)\times P\left(X_{5}=00|X_{1}=01,X_{2}=00\right)\\ & = & f\left(1-f\right)\times {\left(1-f\right)}^{2}\times \frac{1}{2}\times \frac{1}{2}\times \frac{1}{2}=\frac{f{\left(1-f\right)}^{3}}{8} \end{array}$$

However, in practice, the true genotype *X*_*i*_ is almost always either partially observed or not observed at all. Indeed, when a genotyped individual carries the disease mutation, we know that *X*_*i*_ = 11 in the (rare) homozygous case, but we only know that *X*_*i*_ ∈ {10, 01} in the heterozygous case. Similarly, a non genotyped but affected individual only implies that *X*_*i*_ ≠ 00 (since all affected individual are mutation carriers). Moreover, a non genotyped and non affected individual *i* implies that *X*_*i*_ ∈ {00, 01, 10, 11}. Finally, a non carrier genotyped individual implies that *X*_*i*_ = 00 (assuming a 100% sensitivity of the mutation search procedure). Moreover, note that Genotyping errors can easily be added to the model. This uncertainty will be later rigorously taken into account through probabilistic computations using belief propagation in Bayesian networks (see Section “E-step”).

#### Survival part

We recall that ***δ*** ∈ {0, 1}^*n*^ is the censoring indicator. The survival part is defined for any carrier *i* with *X*_*i*_ ≠ 00 as $P\left(T_{i}=t|X_{i}\right)=S\left(t\right)\lambda {\left(t\right)}^{\delta_{i}}$ where λ(*t*) is the hazard function, *S*(*t*) the survival function defined by *S*(*t*) = exp(−Λ(*t*)) and $\Lambda \left(t\right)=\int_{0}^{t}\lambda \left(u\right)du$ the cumulative hazard. Note that for the sake of simplicity, we abusively use the probability symbol P to actually denote a (conditional) density in the case where *δ*_*i*_ = 1. Since non-carrier cannot be affected, they do not appear in the log-likelihood. For simplification purpose it is nevertheless useful to make them appear in the expression with a null contribution by abusively writing:

$$\begin{array}{c} \text{log}\;P\left(T_{i}=t|X_{i}\right)=\{\begin{array}{cc} -\Lambda \left(t\right)+\delta_{i}\;\text{log}\;\lambda \left(t\right) & \text{if}\;X_{i}\neq 00\\ 0 & \text{if}\;X_{i}=00 \end{array}. \end{array}$$

#### Accounting for covariates

Note that covariates can easily be added to the model through a proportional hazard model defining hereafter. Let $Z\in R^{n\times p}$ be the covariate matrix, the model accounting for ***Z*** can be written as follows:

$$\begin{array}{c} \text{log}P\left(T_{i}=t|X_{i}\right)=\{\begin{array}{cc} -\Lambda_{0}\left(t\right)e^{Z_{i}\beta }+\delta_{i}(\text{log}\lambda_{0}\left(t\right)+Z_{i}\beta ) & \text{if}\;X_{i}\neq 00\\ 0 & \text{if}\;X_{i}=00 \end{array} \end{array}$$

where λ_0_(*t*) is the baseline hazard, Λ_0_(*t*) is the baseline cumulative hazard, $Z_{i}\in R^{1\times p}$ the *i*^th^ row of ***Z*** and $\beta \in R^{p\times 1}$ is the proportional effect coefficient.

### The Expectation Maximization algorithm

As stated above, most of the genotypes *X*_*i*_ are not observed at all, and even for the genotyped individuals, we often only have partial information (*e.g*., we cannot distinguish between 01 and 10). We therefore consider the variable ***X*** as a latent variable and denote by X the set of acceptable genotypes (*e.g.* $X_{i}=\left\{00,01,10,11\right\}$ if we have no information on *X*_*i*_, $X_{i}=\left\{01,10\right\}$ if we know that *X*_*i*_ is heterozygous, $X_{i}=\left\{00\right\}$ for a non-carrier, etc.). We denote by “ev” the *evidence* corresponding to all the available information, i.e. the available genotype informations (X∈X) as well as the partially censored ***T***. Note that this notion of ‘evidence’ in Bayesian network context is similar but not exactly the same as the notion of ‘evidence’ in Bayesian statistics. In order to maximize the log-likelihood function of the model in the presence of incomplete data, we use the EM algorithm [10]. To that end, let us introduce the following auxiliary *Q* function:

$$\begin{array}{c} Q(\theta |\theta_{\text{old}})=\sum_{X}P(X|\text{ev};\theta_{\text{old}})\text{log}P\left(X,\text{ev};\theta \right) \end{array}$$

where ***θ*** (resp. ***θ***_old_) contains the current (resp. previous) version of the parametric (proportional effect coefficients) and non-parametric (survival functions) components of the model. Formally, the classical *Q* function of the EM algorithm is equal to the present function plus a constant term in *θ*. Therefore, maximizing our function instead of the original one does not affect our algorithm.

Since the genetic component of the model has no parameter (the allele frequency *f* is supposed to be known and a Mendelian transmission of the alleles is assumed—see Section “Genetic part”), by using the model properties it is straightforward to rewrite the *Q* function as follows

$$\begin{array}{c} Q\left(\theta |\theta_{\text{old}}\right)=\text{cst.}+\sum_{i=1}^{n}\underset{w_{i}}{\underset{︸}{P(X_{i}\neq 00|\text{ev};\;\theta_{\text{old}})}}\text{log}\;P\left(T_{i}|X_{i}\neq 00;\theta \right) \end{array}$$ (1)

Starting from an arbitrary value of ***θ*** = ***θ***_0_, the following two steps are iterated until the estimates converge:

- E-step: for computing the weights $w_{i}=P(X_{i}\neq 00|\text{ev};\;\theta_{\text{old}})$ using ***θ***_old_ = ***θ*** (that are conditional probabilities);
- M-step: for maximizing the *Q* function with respect to ***θ*** and obtaining a new estimate.

#### E-step

In order to compute the conditional probabilities $w_{i}=P(X_{i}\neq 00|\text{ev};\;\theta_{\text{old}})$ it is first necessary to compute their common denominator:

$$\begin{array}{ccc} P(\text{ev};\theta_{\text{old}}) & = & \sum_{X}P\left(X,\text{ev};\theta_{\text{old}}\right)\\ & = & \sum_{X}\{\prod_{i=1}^{n}1_{X_{i}\in X_{i}}P\left(T_{i}|X_{i};\theta_{\text{old}}\right)\prod_{i\in F}P\left(X_{i}\right)\prod_{i\in I\backslash F}P\left(X_{i}|X_{{\text{pat}}_{i}},X_{{\text{mat}}_{i}}\right)\} \end{array}$$

Since ***X*** has 4^*n*^ possible configurations in the worst case, it is clearly impossible to simply enumerate these configurations even for moderate size pedigrees. Therefore, one needs a computationally more efficient approach. When the pedigree has no loop (i.e. the pedigree is a tree), the Elston-Stewart algorithm [17] suggests to eliminate the variables *X*_*i*_ from the above sum-product by *peeling* individuals from the last generations up to the oldest common ancestor. The resulting algorithm has a $O(n\times 4^{3})$ complexity which allows to efficiently handle even large pedigrees as long as they have no loop. However, in practice, it is not rare to encounter loops in pedigree (*e.g.*, consanguinity loops). Fortunately, Elston-Stewart can be adapted to the presence of loops by introducing the notion of *cut-sets* [18] which results in a $O(n\times 4^{k})$ complexity, where *k* ≥ 3 correspond to the size of the largest cut-set in the peeling sequence. Typically *k* = 4 to 6 for most pedigrees, but *k* can also grow very large resulting in intractable exact computations for highly complex pedigrees (*e.g.* inuit pedigree [19]). This cut-set version of Elston-Stewart (as well as variants of Lander-Green [20] for multi-point analysis) is implemented in the well-known Mendel software [21] which can efficiently perform likelihood computations in complex pedigrees.

As pointed out in [22], the distribution of genotypes in pedigree can also be described as a Bayesian network, a model that belongs to a wide class of probabilistic graphical models with strong mathematical background and well-known theory for efficiently performing sum-product computations [11]. The approach consists in sequentially eliminating variables from the graphical model taking into account the clique structures of the corresponding graph. This approach results in the construction of a *junction tree* whose tree-width (size of the largest clique) is precisely equivalent to *k* for cut-sets approaches. These algorithms are called sum-product, message passing, or belief propagation algorithm and they have been used by many authors in the context of genetics [22–26]. One interesting feature of belief propagation in pedigree is that, for the computational cost of two likelihood computation, this approach provides the full posterior distribution of the system, including the marginal posterior distribution of all genotypes (see [11, 22]). But as pointed out by [27], the Elston-Stewart peeling algorithm can be extended to obtain a similar feature. The resulting algorithm is in fact *exactly* the forward/backward equivalent of belief propagation for a peeling sequence (sequence of variable elimination).

In this paper, we use a *ad hoc* low performance R implementation of belief propagation in pedigree called bped (available as supplementary material). At each E-step of the EM algorithm, we provide to this command-line program two files:

1. a pedigree structure file as a classical .ped file;
2. an evidence file containing the evidence $1_{X_{i}\in \text{ev}}P\left(T_{i}|X_{i};\theta_{\text{old}}\right)$ for all *i* ∈ {1, …, *n*} and for all *X*_*i*_ ∈ {00, 01, 10, 11}.

For a non-affected individual (*δ*_*i*_ = 0), one has:

$$\begin{array}{c} P\left(T_{i}|X_{i};\theta_{\text{old}}\right)=\{\begin{array}{cc} S(T_{i}) & \text{if}\;X_{i}\neq 00\\ 1 & \text{if}\;X_{i}=00 \end{array} \end{array}$$

and for an affected individual (*δ*_*i*_ = 1) one has:

$$\begin{array}{c} P\left(T_{i}|X_{i};\theta_{\text{old}}\right)=\{\begin{array}{cc} S\left(T_{i}\right)\lambda \left(T_{i}\right) & \text{if}\;X_{i}\neq 00\\ 0 & \text{if}\;X_{i}=00 \end{array}=S\left(T_{i}\right)\lambda \left(T_{i}\right)\times \{\begin{array}{cc} 1 & \text{if}\;X_{i}\neq 00\\ 0 & \text{if}\;X_{i}=00 \end{array} \end{array}$$

Since the proportion factor *S*(*T*_*i*_)λ(*T*_*i*_) does not depend on *X*_*i*_, its values will not affect in any way the posterior distribution *P*(*X*_*i*_|ev; *θ*_old_). Indeed, since we compute the posterior distribution of all *X*_*i*_, any multiplicative factor that appears in the prior distributions will cancel out in the posterior. This is exactly the case for the *S*(*T*_*i*_)λ(*T*_*i*_) factor which can then be removed. Hence we can replace this proportion factor by 1 and simply use:

$$\begin{array}{c} P\left(T_{i}|X_{i};\theta_{\text{old}}\right)\propto \{\begin{array}{cc} S(T_{i}) & \text{if}\;\delta_{i}=0\;\text{and}\;X_{i}\neq 00\\ 1 & \text{if}\;\delta_{i}=0\;\text{and}\;X_{i}=00\\ 1 & \text{if}\;\delta_{i}=1\;\text{and}\;X_{i}\neq 00\\ 0 & \text{if}\;\delta_{i}=1\;\text{and}\;X_{i}=00 \end{array} \end{array}$$

in the evidence file. It is therefore clear that the knowledge of λ(*t*) is not required for this procedure which is of particular interest since non-parametric survival estimate like Kaplan-Meier usually provides only the expression of *S*(*t*) and not the one of λ(*t*).

Then, bped performs the BP and computes the posterior marginal distribution $P(X_{i}|\text{ev};\;\theta_{\text{old}})$ for all individual *i*, from which the weights $w_{i}=P(X_{i}\neq 00|\text{ev};\;\theta_{\text{old}})$ are derived.

#### M-step

Once the weights *w*_*i*_ have been computed (at the E-step), the model components can be updated by maximizing Eq (1) which is simply a weighted survival log-likelihood function where each individual observation receives the weight *w*_*i*_. Since most statistical softwares allow for weighted observations, we can therefore rely on well-established existing survival tools for performing our M-step. Using the programming software R [13], we can for example take advantage of the robust survival package [12, 28] which provides non-parametric Kaplan-Meier estimation of the survival through the survfit() function. Note that the coxph() can also be combined with survfit() to provide non-parametric Nelson-Aalen survival estimates taking into account proportional hazard effects. In addition, using full parametric survival estimation procedures, such as the survreg() function, allows the method to provide alternative classical survival estimation (namely Weibull, exponential, Gaussian, logistic, log-normal, log-logistic) with no additional development costs. Even if the primary purpose and novelty of our method is to provide non-parametric survival estimate, the possibility to fit classical parametric survival estimates is also an interesting feature especially considering that few or none of the previously published methods provide any practical implementation.

#### Practical implementation

EM initialization is performed by affecting random weights *w*_*i*_ to all individuals in each pedigree (*e.g.*, drawn from a uniform distribution on [0, 1] and normalized to ensure the sum-to-one constraint). Then, a first M-step is performed using these weights in order to provide an initial value of ***θ***. The EM iterations are run until numerical convergence is achieved. The usual convergence criterion is such that the absolute error between survival estimates (*e.g.*, baseline survival at age 20, 40, 60, 80) decreases below a threshold (*e.g.*, 10^−10^) between two consecutive iterations of the algorithm. The 95% pointwise confidence intervals are simply provided by the standard (weighted) Kaplan-Meier (or Nelson-Aalen if we consider covariates) estimation of the survival.

## 1 Validation on simulated datasets

For validation purposes we first consider the application of our method on simulated datasets. In order to simulate realistic pedigree structure (parental relationships and individual genders), we use 64 French and Portugese hATTR families from [2] totalizing 1,095 individuals. These 64 families were replicated three times resulting in a dataset of *n* = 3,285 individuals in 192 families. Genotypes were assigned using the Hardy-Weinberg distribution for the founders and respecting the Mendelian transmission for the non-founders. We have used an allele frequency of *f* = 0.20 in order to obtain enough informative families (without simulating any ascertainment process). The gender of the transmitting parent was not taken into account in this work (no distinction between *X* = 01 and *X* = 10). Thus, the genotype of individual *i* was binary and individual *i* was a mutation carrier if *X*_*i*_ ∈ {01, 10, 11} and non carrier if *X*_*i*_ = 00. The age at diagnosis was simulated according to a piecewise constant hazard rate function, λ(*t*), given as follows:

$$\lambda \left(t\right)=\{\begin{array}{cc} 0 & \;\text{if}\;t\in [0,20]\\ 0.02 & \;\text{if}\;t\in ]20,40]\\ 0.10 & \;\text{if}\;t\in ]40,60]\\ 0.05 & \;\text{if}\;t>60 \end{array}.$$

A uniform censoring data between 15 and 80 years resulting in a censoring rate of roughly 30% (similar to real data censoring rates) was simulated. A total of 10% of the individuals (uniformly selected) was supposed to be genotyped (without error) while the 90% remaining individuals were not.

**Fig 1:**
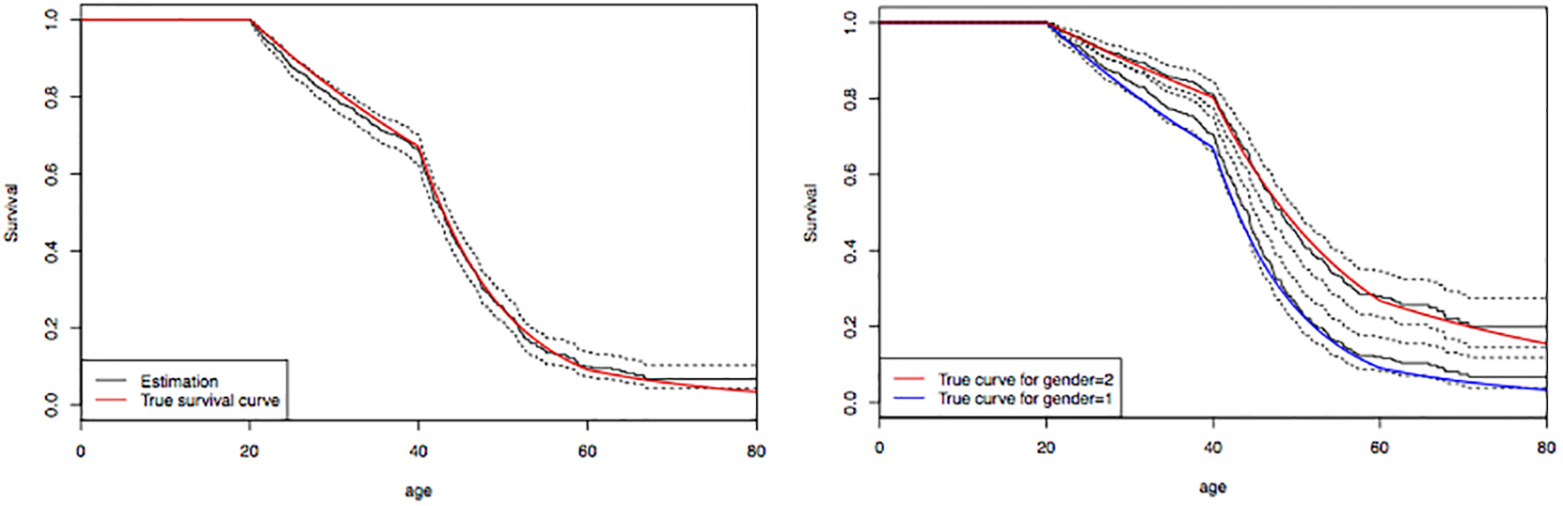
Simulated dataset. Reference and estimation of the survival function *S*(*t*) for carriers with 95% point-wise confidence intervals (dashed lines). A total of *n* = 3,285 (1,641 males and 1,644 females) individuals including 441 affected and 319 genotyped. Left: simulation and estimation without gender effect. Right: simulation and estimation with a proportional protective effect for females (gender = 2).

One can see on Fig 1 (left) the non-parametric Kaplan-Meier estimation obtained at the end of the EM algorithm. Despite the fact that only 10% of the individuals where genotyped, the method clearly manages to provide accurate estimates. Unsurprisingly, the size of the confidence intervals decrease when the sample or the number of affected individuals increases (data not shown).

In order to demonstrate the ability of our method to deal with semi-parametric estimation (non-parametric baseline survival and proportional hazard) we now consider the previous incidence λ(*t*) as a baseline incidence which is also the male incidence. Moreover, we assume that the females benefit from a protective effect and we use the relative hazard (RH) 0.55 = exp(−0.6) (which means that males have an instantaneous risk 1.8 higher than females). We denote by *β* = −0.6 the regression parameter. In our simulation, we hence generate the time to diagnosis with the survival *S*_1_(*t*) = exp(−Λ(*t*)) for males and with the survival *S*_2_(*t*) = exp(−Λ(*t*)*e*^*β*^) for females. Censoring and genotyping remain unchanged.

Covariates can be taken into account by stratifying on these covariates. However, since proportional hazard models are commonly considered in this context, we also perform a simulation where we assume a PH effect of the gender.

At each M-step of the EM algorithm we fit both a Cox PH model using gender as factor (gender = 1 as default) and then perform a non-parametric (Nelson-Aalen) estimation of the baseline survival. At the end of the algorithm, estimation of the proportional effect can be combined with the baseline survival estimation to provide survival estimations for the two classes. Alternatively, a purely stratified approach is also possible and give very similar results (data not shown) but since our purpose was here to illustrate the semi-parametric approach, we only give its results. The final Cox fitting gives that the *β* parameter was estimated by $\hat{\beta }=-0.59$ (p-value < 0.01) which is very close to the true value *β* = −0.6, and one can see on Fig 1 (right) the survival estimates for the two classes. Like for the simpler case with no covariates, the estimations are quite consistent with the ground truth. Again, increasing the sample size or the number of affected individuals leads to sharper confidence intervals (data not shown).

Now that the method appears to be validated on simulated datasets, we can consider real datasets.

## 2 Application to the hATTR

In this section the proposed method is applied to the transthyretin hereditary amyloidosis (hATTR), a severe autosomal dominant disorder caused by a mutation of the transthyretin (TTR) gene. The disorder initially described in Portugal is now recognized across the world with areas of highest prevalence like in Sweden or in Japan [29]. The ATTR-Val30Met (denoted MET30 from now on) is the most frequent pathogenic variant in Europe and virtually the only one detected in Portugal and Sweden. For this particular variant, a wide range of age at onset is observed with an average 30 (resp. 56) in Portuguese (resp. Swedish) families.

In France, the population of hATTR is heterogeneous including families from Portuguese descent presenting alike those from Portugal and families from French descent. The latter are characterized by a heterogeneity of pathogenic TTR variants, including the MET30 in 40% and a later onset of symptoms averaging 58 years of age. Fortunately, significant therapeutic advances occurred in the recent years with the aim to stabilize the disease progression. In this setting, a better knowledge of the risk of being symptomatic for carrier is highly needed to guide their follow up and to manage patients at the very onset of symptoms. It may also give clues on our understanding of the pheno-genotypic variability observed.

Because of the low allelic frequency, random sampling is not a tractable approach to obtain informative samples. As a consequence, data are usually obtained from families ascertained through affected individuals. Indeed, as all affected individuals necessarily carry the mutation, families ascertained in this way are very informative for estimating survival function. The drawback of this procedure is that the survival function can be significantly overestimated if the ascertainment process is not taken into account [30]. Therefore, an adjustment for the ascertainment bias is required. Different adjustments for ascertainment bias have already been proposed in order to provide valid risk estimates of a genetic disease (see for instance [1, 3, 6]). In these applications, the ascertainment bias was corrected by a classical method that consists in simply removing the phenotypic information of the individual (called *proband*) who allowed his family to be selected. This ascertainment correction is a well-known (and validated) preprocessing technique whose relevance is not discussed here.

Here we considered three datasets (see Table 2): the French dataset totalized 46 families from French descent with as many as 12 different pathogenic TTR variants including the MET30 in 22; the Portuguese dataset included 33 MET30 families from Portugal; the 3rd dataset enrolled 77 MET30 kindreds from Northern Sweden. These data have been described in two previously published studies [2, 31]. Both studies were approved by local ethic commitees (EC) in France and in Sweden, respectively. In this setting, as required by the EC and stated in the two publications, geno-phenotypic information on families have to remain anonymous for ethical and medical reasons and cannot be disclosed.

**Table 2:** The three hATTR datasets.

Dataset	French	Portuguese	Swedish
number of families	46	33	77
number of individuals	624	384	1,353
number of affected	115	122	230
known genotypes	58.3%	60.8%	24.8%

The frequency of mutated allele was set to *f* = 0.001 [6, 32]. This parameter is generally unknown in practice. In addition, it has been shown in [6] that the survival estimations are not highly sensitive to this parameter.

For each dataset, we provide a semi-parametric survival estimation with a gender proportional hazard effect. We provide p-values for the gender effect through Cox’s (partial-) likelihood ratio tests. For each dataset, the results are compared to previously published analyses.

**Fig 2:**
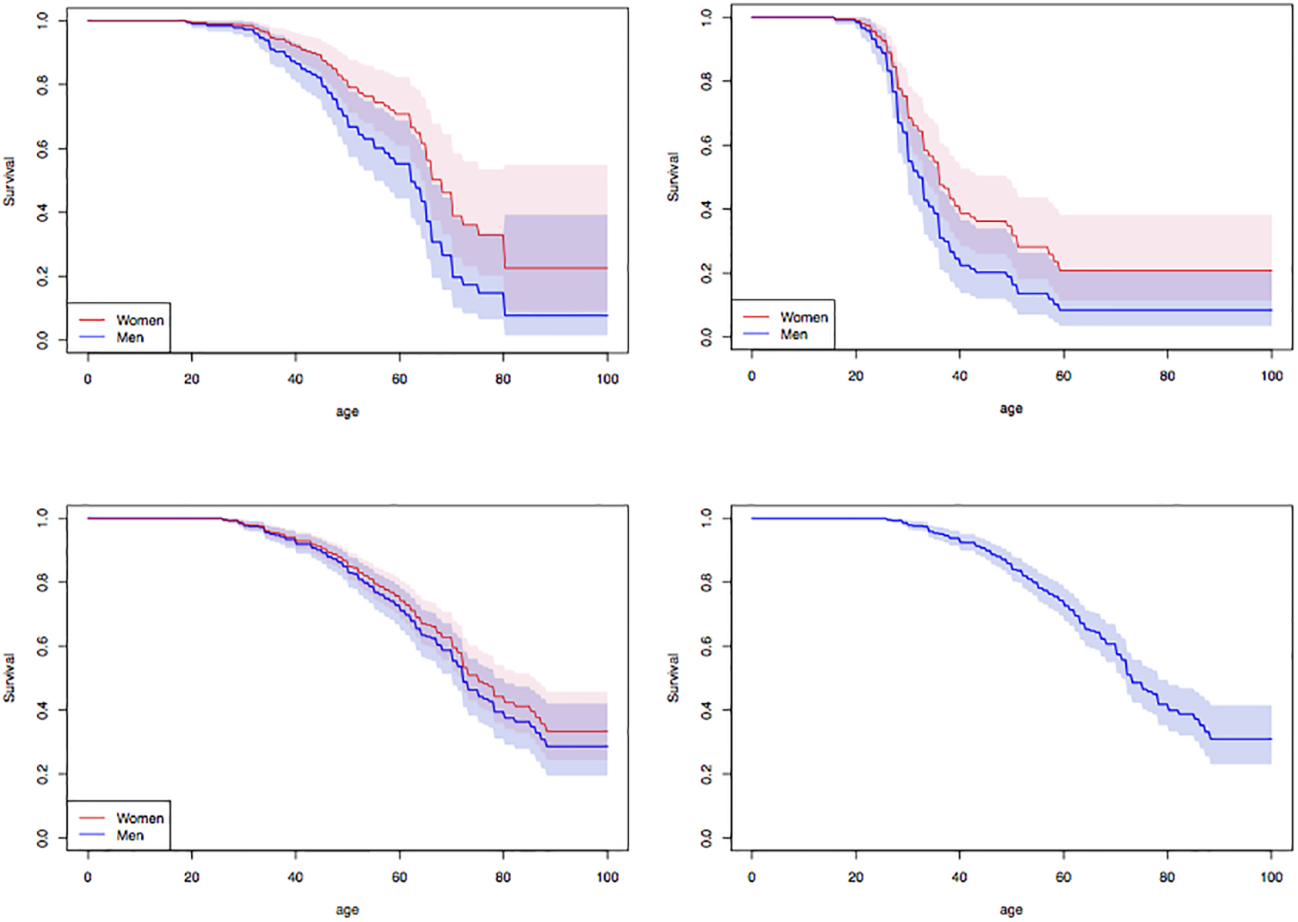
Survival estimates. Top-Left: French dataset with a gender PH effect (RH 1.7, Cox’s p-value 0.030); Top-Right: Portugese dataset with a gender PH effect (RH 1.57, Cox’s p-value 0.033); Bottom-Left: Swedish dataset with a non-significant gender PH effect (Cox’s p-value 0.42). Bottom-Right: Swedish dataset without any gender PH effect. 95% point-wise confidence intervals are given by the colored regions.

Fig 2 shows the survival estimates by gender for the three datasets. For the French dataset (top-left Fig 2), one observes a later disease onset (median around 70) than in the Portuguese sample (Fig 2, top-right) showing a median around age 45 years. A significantly higher instantaneous risk is observed for men compared to women in both the French (RH 1.7, Cox’s p-value 0.03) and the Portuguese (RH 1.57, Cox’s p-value 0.033) datasets. In contrast, we found no gender effect in the Swedish dataset (Fig 2, bottom-left, Cox’s p-value 0.42) and hence present the estimate without gender effect in Fig 2 (bottom-right). The disease onset appears to be much later in the Swedish population in comparison with the French and Portugese populations.

These observations are highly consistent with the previously published analyses [2, 31]. In the previous stratified analysis, the gender difference was found lower and not significant in the whole French dataset. This difference can be explained by the additional power provided by the proportional hazard model used here. For comparison purposes we fitted on the French data a stratified non-parametric survival and tested for difference between genders using the log-rank test resulting in a non significant p-value of 0.122, which is consistent with the previous study. The previously reported heterogeneity in age of onset across the three datasets is confirmed in the present study.

## 3 Discussion

In the present article we introduced a flexible and robust framework to estimate survival function from familial data in cases of age-dependent genetic diseases. Our new method provides a unifying way to simply implement both previously published methods (parametric Weibull-based) as well as new interesting extension such as the non-parametric or semi-parametric extensions.

In order to tackle the challenging problem of the unknown genotypes in the family data, our method relies on the EM algorithm and decomposes the problem into two steps: the E-step which uses belief propagation in Bayesian networks to compute marginal individual posterior carrier distribution, and the M-step which estimates survival using weighted observations.

The key feature of our approach is that these two steps are handled by robust and validated implementations: the bped command-line program for the belief propagation, and the survival package (statistical software R [13]) for the survival estimates. We can therefore consider any baseline survival estimators, either parametric (*e.g.*, Weibull, exponential, log normal, etc.) or non parametric (Kaplan-Meier). Moreover, these estimators can be easily combined with Cox’s proportional hazard models and with stratification.

Note that in the present paper we focused on the particular case where non-carriers cannot be affected (survival of 1.0) and where the genetic model is dominant. However, the method can be easily extended to more general models (sporadic cases, recessive model, etc.) as long as the incidence among non-carriers is known (i.e. estimated from the general population). Moreover, more complex models allowing for genotyping errors or even pedigree errors (for instance wrong filiation) can be incorporated, as done in [33], even if, in the present work, we have focused on the most basic (but reasonable) model.

In the application part, as pedigrees are ascertained through an affected individual, the proband’s phenotype exclusion method is used to avoid ascertainment bias. However, other ascertainment corrections can be used if the ascertainment process is more complex (*e.g.*, ascertainment on family criteria in a complex disease with monogenic sub-entities, such as breast and ovarian cancers with the BRCA mutations). Again, this is in favor of the flexibility of the proposed method.

Concerning the perspectives, an interesting extension of this work would be to account for a possible correlation between members of the same family by including a frailty in the survival function. The familial frailty would typically represent an unknown shared exposure to some environmental factors or to some kinds of polygenic effect. However, the estimation of such models is known to be challenging, especially in the context of non-parametric survival estimation (see *e.g.*, [34, 35]). Further investigations will be conducted on this important topic in a forthcoming work. However, in this work and particularly for applications to monogenic diseases (such as hATTR), this frailty aspect should not modify the estimation results. Moreover, the proposed method allows to take into account the parent of origin effect. Thus, it would be very interesting to study the robustness of the survival function estimation when the parent-of-origin effect is analyzed.

## Supporting information

S1 File R source code demo. (DOCX)

S2 File CPP-recueil-data. (PDF)
