# Supplementary material for: Non-parametric estimation of survival in age-dependent genetic disease and application to the transthyretin-related hereditary amyloidosis
Source: PLoS One. 2018 Sep 25;13(9):e0203860. doi: 10.1371/journal.pone.0203860 (PMC6155453; doi:10.1371/journal.pone.0203860)
Supplement: S1 File — (DOCX) [file pone.0203860.s001.docx]

R demo source code

0.6

0.8

*G. Nuel 05 juin, 2018*

# Survival Estimate with Known Genotypes

1.0

*#load("ped.Rdata")*

idx=(ped$geno>0) fit=**survfit**(**Surv**(ped$time[idx],ped$status[idx])~1) **plot**(fit)

**lines**(**c**(0,20:80),**c**(1,**pexp**(0:60,rate=1/20,lower.tail=FALSE)),col="red") **legend**("topright",**c**("Kaplan-Meier","True Survival"),col=1:2,lty=1,bg="white")

## 0 10 20 30 40 50 60 70

Kaplan−Meier True Survival

0.0

0.2

0.4

**Survival Estimate with Unknown Genotypes**

n=**nrow**(ped)

*# random initialization*

weights=**runif**(n)

*# but all affected must be carriers* affected=(ped$status==1) weights[affected]=1.0

*# main loop*

for (iter in1:50) {

*# weight Kaplan-Meier fit* fit=**survfit**(**Surv**(ped$time,ped$status)~1,weights=weights) *# retrieve survival and hazard as step functions* S=**stepfun**(fit$time,**c**(1.0,fit$surv))

*# verbose output*

**cat**("iter=",iter,"S(30)=",**S**(30),"S(50)=",**S**(50),"S(70)=",**S**(70),"\n")

*# build evidence*

ev=**matrix**(NA,n,4)

ev[affected,1]=0.0; ev[affected,2]=1.0;

ev[!affected,1]=1.0; ev[!affected,2]=**S**(ped$time[!affected]); ev[,3]=ev[,4]=ev[,2]

*# perform belief propagation* res=**bped**(bn,ev) post=res$marginal weights=**apply**(post[,-1],1,sum)

}

## iter= 1 S(30)= 0.8132826 S(50)= 0.6236955 S(70)= 0.5063622

## iter= 2 S(30)= 0.6880188 S(50)= 0.3815691 S(70)= 0.2065157

## iter= 3 S(30)= 0.6328908 S(50)= 0.2752908 S(70)= 0.07532941

## iter= 4 S(30)= 0.6072951 S(50)= 0.2263406 S(70)= 0.02325502

## iter= 5 S(30)= 0.5957437 S(50)= 0.2043845 S(70)= 0.006118727

## iter= 6 S(30)= 0.5908839 S(50)= 0.1952001 S(70)= 0.001455062

## iter= 7 S(30)= 0.5890001 S(50)= 0.1916583 S(70)= 0.0003301826

## iter= 8 S(30)= 0.5883192 S(50)= 0.1903835 S(70)= 7.358552e-05

## iter= 9 S(30)= 0.5880854 S(50)= 0.1899472 S(70)= 1.629639e-05

| ## | iter= | 10 | S(30)= | 0.5880079 | S(50)= | 0.1898028 | S(70)= | 3.601468e-06 |
| --- | --- | --- | --- | --- | --- | --- | --- | --- |
| ## | iter= | 11 | S(30)= | 0.5879828 | S(50)= | 0.1897562 | S(70)= | 7.953769e-07 |
| ## | iter= | 12 | S(30)= | 0.5879748 | S(50)= | 0.1897414 | S(70)= | 1.756195e-07 |
| ## | iter= | 13 | S(30)= | 0.5879723 | S(50)= | 0.1897367 | S(70)= | 3.87742e-08 |
| ## | iter= | 14 | S(30)= | 0.5879715 | S(50)= | 0.1897352 | S(70)= | 8.560591e-09 |
| ## | iter= | 15 | S(30)= | 0.5879713 | S(50)= | 0.1897348 | S(70)= | 1.89e-09 |
| ## | iter= | 16 | S(30)= | 0.5879712 | S(50)= | 0.1897346 | S(70)= | 4.172718e-10 |
| ## | iter= | 17 | S(30)= | 0.5879712 | S(50)= | 0.1897346 | S(70)= | 9.212467e-11 |
| ## | iter= | 18 | S(30)= | 0.5879712 | S(50)= | 0.1897346 | S(70)= | 2.033915e-11 |
| ## | iter= | 19 | S(30)= | 0.5879712 | S(50)= | 0.1897346 | S(70)= | 4.490449e-12 |
| ## | iter= | 20 | S(30)= | 0.5879712 | S(50)= | 0.1897346 | S(70)= | 9.914014e-13 |
| ## | iter= | 21 | S(30)= | 0.5879712 | S(50)= | 0.1897346 | S(70)= | 2.188797e-13 |
| ## | iter= | 22 | S(30)= | 0.5879712 | S(50)= | 0.1897346 | S(70)= | 4.832352e-14 |
| ## | iter= | 23 | S(30)= | 0.5879712 | S(50)= | 0.1897346 | S(70)= | 1.066522e-14 |
| ## | iter= | 24 | S(30)= | 0.5879712 | S(50)= | 0.1897346 | S(70)= | 2.356713e-15 |
| ## | iter= | 25 | S(30)= | 0.5879712 | S(50)= | 0.1897346 | S(70)= | 5.237139e-16 |
| ## | iter= | 26 | S(30)= | 0.5879712 | S(50)= | 0.1897346 | S(70)= | 1.200178e-16 |
| ## | iter= | 27 | S(30)= | 0.5879712 | S(50)= | 0.1897346 | S(70)= | 2.182141e-17 |
| ## | iter= | 28 | S(30)= | 0.5879712 | S(50)= | 0.1897346 | S(70)= | 0 |
| ## | iter= | 29 | S(30)= | 0.5879712 | S(50)= | 0.1897346 | S(70)= | 0 |
| ## | iter= | 30 | S(30)= | 0.5879712 | S(50)= | 0.1897346 | S(70)= | 0 |
| ## | iter= | 31 | S(30)= | 0.5879712 | S(50)= | 0.1897346 | S(70)= | 0 |
| ## | iter= | 32 | S(30)= | 0.5879712 | S(50)= | 0.1897346 | S(70)= | 0 |
| ## | iter= | 33 | S(30)= | 0.5879712 | S(50)= | 0.1897346 | S(70)= | 0 |
| ## | iter= | 34 | S(30)= | 0.5879712 | S(50)= | 0.1897346 | S(70)= | 0 |
| ## | iter= | 35 | S(30)= | 0.5879712 | S(50)= | 0.1897346 | S(70)= | 0 |
| ## | iter= | 36 | S(30)= | 0.5879712 | S(50)= | 0.1897346 | S(70)= | 0 |
| ## | iter= | 37 | S(30)= | 0.5879712 | S(50)= | 0.1897346 | S(70)= | 0 |
| ## | iter= | 38 | S(30)= | 0.5879712 | S(50)= | 0.1897346 | S(70)= | 0 |
| ## | iter= | 39 | S(30)= | 0.5879712 | S(50)= | 0.1897346 | S(70)= | 0 |
| ## | iter= | 40 | S(30)= | 0.5879712 | S(50)= | 0.1897346 | S(70)= | 0 |
| ## | iter= | 41 | S(30)= | 0.5879712 | S(50)= | 0.1897346 | S(70)= | 0 |
| ## | iter= | 42 | S(30)= | 0.5879712 | S(50)= | 0.1897346 | S(70)= | 0 |

## iter= 43 S(30)= 0.5879712 S(50)= 0.1897346 S(70)= 0

## iter= 44 S(30)= 0.5879712 S(50)= 0.1897346 S(70)= 0

## iter= 45 S(30)= 0.5879712 S(50)= 0.1897346 S(70)= 0

## iter= 46 S(30)= 0.5879712 S(50)= 0.1897346 S(70)= 0

## iter= 47 S(30)= 0.5879712 S(50)= 0.1897346 S(70)= 0

## iter= 48 S(30)= 0.5879712 S(50)= 0.1897346 S(70)= 0

## iter= 49 S(30)= 0.5879712 S(50)= 0.1897346 S(70)= 0

## iter= 50 S(30)= 0.5879712 S(50)= 0.1897346 S(70)= 0

**plot**(fit) **lines**(**c**(0,20:80),**c**(1,**pexp**(0:60,rate=1/20,lower.tail=FALSE)),col="red")

**legend**("topright",**c**("EM Kaplan-Meier","True Survival"),col=1:2,lty=1,bg="white")

0.6

0.8

1.0

## 0 20 40 60 80

carrier=(ped$geno>0)

**plot**(weights,col=carrier+1,pch=carrier+1)

**legend**("right",**c**("non-carrier","carrier"),col=1:2,pch=1:2,bg="white")

EM Kaplan−Meier True Survival

0.0

0.2

0.4

Sensitivity

0.4

0.6

0.6

0.8

1.0

0 50 100 150


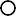

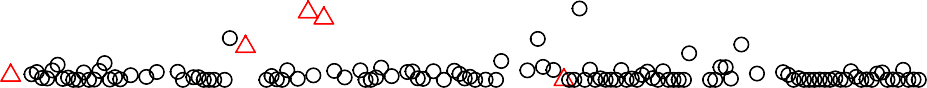

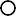

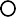

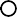

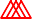

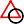

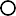


non−carrier carrier

weights

0.0

0.2

0.4

Index

**require**(pROC) res=**roc**(cases=weights[carrier],controls=weights[!carrier],ci=TRUE) **plot**(res)

0.8

1.0

1.0 0.5 0.0

0.0

0.2

Specificity
