# Supplementary material for: Non-parametric estimation of survival in age-dependent genetic disease and application to the transthyretin-related hereditary amyloidosis
Source: PLoS One. 2018 Sep 25;13(9):e0203860. doi: 10.1371/journal.pone.0203860 (PMC6155453; doi:10.1371/journal.pone.0203860)
Supplement: S2 File — (PDF) [file pone.0203860.s002.PDF]

Comité Consultatif de Protection des Personnes dans la Recherche Biomédicale de Bicêtre - Hôpital Bicêtre - 78 rue du Général Leclerc -
94270 KREMLIN BICETRE Tél. 01.45.21.28.46 - Fax 01.45.21.21.45 -
Président : Pr Marcel HAYAT - Secrétaire : Mme PILATE

Projet de Recherche N° : ***98-46***

Kremlin Bicêtre le 9 juillet 1998

Le Comité a été saisi le 4 juin 1998

par Madame le Docteur PLANTE BORDENEUVE
concernant le projet de recherche ***sans bénéfice individuel direct*** et intitulé
" **ETUDE GENETIQUE CLINIQUE ET MOLECULAIRE DES NEUROPATHIES AMYLOIDES HEREDITAIRES EN FRANCE**"
(Référence du promoteur P980103)

dont le promoteur est : ASSISTANCE PUBLIQUE - HOPITAUX DE PARIS
3 avenue Victoria
75004 PARIS

Le comité a examiné les informations relatives à ce projet [1] lors des séances du 9 juin 1998 et 7 juillet 1998

Membres présents lors de la délibération de votre protocole
Mmes C. ASTOUL Infirmière dans un C.H.R.U, M. BOULEY Pharmacien dans un C.H.R.U.,J. GAUDIN Psychologue dans un cabinet privé, M.C. GEORGE Magistrat, C. HENRY Assistante sociale dans un C.H.R.U., A. LAPLANCHE Médecin Spécialisé dans un Centre anti-cancéreux, M. LE MEUR Assistante Sociale, D. SALVAT Infirmière dans un C.H.R.U et A.M. TABURET Pharmacien dans un C.H.U. Mrs G. BARON Pharmacien dans un C.H.R.U.,R. de BEAUREPAIRE Médecin spécialisé, R. DUCARRE Médecin Généraliste, T. EHRHARD Médecin généraliste, D. FETEANU Médecin spécialisé dans un C.H.R.U., P.FRANCOIS Médecin Spécialisé, M. HAYAT Médecin spécialisé dans un centre anti-cancéreux, E. MARTIN représentant du courant éthique, M. PUCHEAULT médecin spécialisé dans un C.H.R.U., J. F. ZAZZO Médecin spécialisé dans un C.H.R.U.

Le Comité a adopté la délibération suivante :

AVIS FAVORABLE sans restriction

Professeur Marcel HAYAT
Président du C.C.P.P.R.B.

1 *Le Comité rappelle à l'investigateur qu'avant la mise en oeuvre, d'un projet* le promoteur transmet à l'autorité administrative compétente une lettre d'intention (art L 209-12 4e alinéa) et souligne les sanctions pénales prévues par les articles L 209-19, L 290-20 et L 209-21 du code de la santé publique pour toute personne ayant pratiqué ou fait pratiquer une recherche en infraction avec ladite loi.
